# Adolescent Cannabis Use and Risk of Psychotic, Bipolar, Depressive, and Anxiety Disorders

**DOI:** 10.1001/jamahealthforum.2025.6839

**Published:** 2026-02-20

**Authors:** Kelly C. Young-Wolff, Catherine A. Cortez, Stacey E. Alexeeff, Lynn D. Silver, Rosalie Liccardo Pacula, Natalie E. Slama, Alisa A. Padon, Derek D. Satre, Cynthia I. Campbell, Maria T. Koshy, Monique B. Does, Stacy A. Sterling

**Affiliations:** 1 Division of Research, Kaiser Permanente Northern California, Pleasanton; 2 Department of Psychiatry and Behavioral Sciences, University of California, San Francisco, San Francisco; 3 Public Health Institute, Oakland, California; 4 Department of Epidemiology and Biostatistics, University of California, San Francisco, San Francisco; 5 Institute for Addiction Science, University of Southern California, Los Angeles,; 6 Leonard D. Schaeffer Center for Health Policy and Economics, Sol Price School of Public Policy, University of Southern California, Los Angeles; 7 The Permanente Medical Group, Kaiser Permanente Northern California, Pleasanton

## Abstract

**Importance:**

As cannabis becomes more accessible and socially accepted, concerns have grown about its potential implications for adolescent mental health. While prior research has linked adolescent cannabis use to psychiatric symptoms, few large, population-based, longitudinal studies have examined associations with clinically diagnosed psychiatric disorders.

**Objective:**

To evaluate whether adolescent cannabis use is associated with an increased risk of incident psychotic, bipolar, depressive, and anxiety disorders during adolescence and young adulthood.

**Design, Setting, and Participants:**

This cohort study included adolescents aged 13 to 17 years who were screened for past-year cannabis use at Kaiser Permanente Northern California from 2016 to 2023. Adolescents were followed up through age 25 years or until December 31, 2023. Data were analyzed from February 21, 2024, to August 27, 2025.

**Exposure:**

Time-varying self-reported past-year cannabis use based on universal, confidential screening during standard pediatric care.

**Main Outcomes and Measures:**

Incident clinician-diagnosed psychotic, bipolar, depressive, and anxiety disorders, which were identified through electronic health records using *International Classification of Disease* codes. Cox proportional hazards regression models were used to measure the strength of associations between adolescent cannabis use and incident psychiatric diagnoses, with adjustments for sex, race and ethnicity, neighborhood deprivation index, insurance type, and time-varying alcohol and other substance use.

**Results:**

Of 463 396 adolescents (234 114 males [50.5%]; mean [SD] age, 14.5 [1.3] years) included in the sample, 136 708 were Hispanic individuals (29.5%), 93 737 were non-Hispanic Asian individuals (20.2%), 35 346 were non-Hispanic Black individuals (7.6%), 153 102 were non-Hispanic White individuals (33.0%), and 18 795 individuals were multiracial or of other races or ethnicities (4.1%). At baseline, 26 345 adolescents (5.7%) self-reported past-year cannabis use. Past-year cannabis use was associated with an increased risk of incident psychotic (adjusted hazard ratio [AHR], 2.19; 95% CI, 1.97-2.42), bipolar (AHR, 2.01; 95% CI, 1.82-2.22), depressive (AHR, 1.34; 95% CI, 1.30-1.39), and anxiety disorders (AHR, 1.24; 95% CI, 1.21-1.28). The strength of the associations between cannabis use and incident depressive and anxiety disorders decreased as adolescents aged. This pattern was similar but slightly attenuated after additional adjustment for past psychiatric conditions (psychotic disorder: AHR, 1.92; 95% CI, 1.73-2.13; bipolar disorder: AHR, 1.73; 95% CI, 1.57-1.90; depressive disorder: AHR, 1.33; 95% CI, 1.29-1.38; anxiety disorder: AHR, 1.19; 95% CI, 1.16-1.23).

**Conclusions and Relevance:**

This cohort study found that adolescent cannabis use was associated with increased risk of incident psychiatric disorders, particularly psychotic and bipolar disorders. These results could inform the development of clinical and educational interventions for parents, adolescents, and clinicians, as well as protective policies to prevent or delay adolescent cannabis use in the context of expanding cannabis legalization.

## Key Points

**Question:**

Is adolescent cannabis use associated with an increased risk of developing psychiatric disorders by young adulthood?

**Findings:**

In this cohort study of 463 396 adolescents aged 13 to 17 years who were universally screened for cannabis use, past-year cannabis use was associated with a significantly increased risk of incident psychotic, bipolar, depressive, and anxiety disorders by age 26 years.

**Meaning:**

This study found that adolescent cannabis use is associated with increased risk of psychiatric disorders in adolescence and young adulthood, highlighting the importance of early prevention efforts, effective public health messaging, and policy development to limit youth exposure as cannabis legalization expands.

## Teaser

This cohort study assesses the association of past-year cannabis use by adolescents with the risk of incident psychiatric, bipolar, depressive, and anxiety disorders by age 26 years.

## Introduction

Cannabis is the most commonly used illicit drug among US adolescents, with 11.2% of adolescents aged 12 to 17 years reporting past-year cannabis use in 2023.^1^ As cannabis becomes increasingly accessible and its use becomes normalized, concerns are growing about its implications for adolescent mental health.^2^ Cannabis use commonly begins during adolescence when many psychiatric disorders first emerge.^3^ Adolescent cannabis use has been associated with psychotic, bipolar, depressive, and anxiety disorders, with varying strength of evidence for each disorder.

Evidence for an association between cannabis and incident psychiatric disorders has been reported, particularly for psychotic disorders.^4,5^ Several longitudinal studies have found that cannabis use in early adolescence, daily use, cannabis use disorder (CUD), or use of higher strength products, is associated with increased risk of developing schizophrenia and other psychotic disorders.^4,6,7^ However, not all studies report that cannabis is a factor in increased risk of these disorders.^8^ Other research has found that cannabis use during adolescence may be associated with an increased risk for hypomanic symptoms in early adulthood, with more frequent use conferring the greatest risk.^9^ Furthermore, studies have found that adolescents and adults with cannabis use or a CUD have a greater risk of developing a bipolar disorder diagnosis, with greatest risk seen immediately after CUD diagnosis.^10,11^ Meta-analyses and systematic reviews have also found evidence suggesting a modest causal relationship between adolescent cannabis use and depression in young adulthood,^2,12^ with the greatest risk among those who heavily used cannabis, while results for risk of anxiety disorders have been less consistent.^2,13^

Despite these potential associations, important questions remain about how adolescent cannabis use relates to the onset of physician-diagnosed psychiatric disorders in large populations. Much of the existing research has focused on small samples, CUD (vs cannabis use), subclinical psychiatric symptoms, or single outcomes in adults or combined samples of adolescents and adults. Few US studies have examined multiple diagnosed psychiatric disorders in a large, diverse, longitudinal cohort of adolescents while accounting for key covariates and changes in cannabis use over time.^4,8,14^

Understanding modifiable factors that precede the development of psychiatric disorders during adolescence and young adulthood is critical to inform more targeted screening, prevention, and intervention strategies. To address these gaps, this retrospective cohort study used data from a large, integrated health care system with universal substance use screening during standard pediatric care to analyze whether adolescent cannabis use was associated with the incidence of 4 separate psychiatric outcomes (psychotic, bipolar, depressive, and anxiety disorders) before age 26 years.

## Methods

### Setting

This cohort study took place in Kaiser Permanente Northern California (KPNC), an integrated health care delivery system providing health care to more than 4.6 million members.^15^ The Teen Well-Check Questionnaire (TWCQ), a comprehensive universal self-administered health-related screening questionnaire, was developed and implemented in the electronic health record (EHR) by KPNC Pediatrics clinical leadership. The TWCQ is administered to adolescents aged 13 to 17 years at biennial well-child visits during standard pediatric care. The KPNC Institutional Review Board approved this study and waived informed consent because study procedures meet Health Insurance Portability and Accountability Act requirements and 42 CFR Part 2 regarding medical records, and all KPNC members are informed upon enrollment in the health plan that their data may be used for research. We followed the Strengthening the Reporting of Observational Studies in Epidemiology (STROBE) reporting guideline.^16^

### Cohort

This retrospective cohort comprised 463 598 adolescents aged 13 to 17 years who completed the TWCQ during well-child visits from January 1, 2016, to December 31, 2023. Eligibility criteria included KPNC enrollment at the time of their well-child visit and a response to the question about past-year cannabis use. To examine the incidence of each psychiatric disorder, adolescents with each outcome diagnosis occurring any time before or on the index date (the first cannabis screening date in the study period) were excluded from the analysis of that outcome; therefore, the sample sizes varied for each outcome. History of *International Classification of Diseases, Ninth Revision* (*ICD-9*) and *International Statistical Classification of Diseases and Related Health Problems, Tenth Revision* (*ICD-10*) psychiatric disorders were identified using all available EHR data prior to baseline, with a maximum lookback period of 17 years (median [IQR], 2.8 [1.2-5.3] years). The 202 adolescents (<0.1%) with a history of all 4 disorders at baseline were excluded (eMethods in Supplement 1).

### Cannabis Exposure and Psychiatric Outcomes

Past-year cannabis use was defined based on response to the question: “During the past year, did you use marijuana?” (yes or no) on the TWCQ completed by the adolescent in a confidential manner during each well-child visit (recommended at least every 2 years). The TWCQ was not completed at other types of visits (eg, acute visit for illness). The exposure was modeled as a time-varying covariate and updated each time that the question was answered at subsequent well-child visits. Incident psychiatric disorders were defined by *ICD-10-Clinical Modification* (*CM*) diagnostic codes from January 2016 to December 2023 and categorized as psychotic, bipolar, depressive, and anxiety disorders (eMethods in Supplement 1).

Covariates included age at well-child visit, sex (female, male, or unknown), self-reported race and ethnicity (Hispanic, non-Hispanic Asian, non-Hispanic Black, non-Hispanic White, and multiracial or other race and ethnicity [including American Indian, Alaska Native, Pacific Islander, Native Hawaiian]), US Census-based neighborhood deprivation index (NDI, categorized into quartiles based on the KPNC population), and insurance type (Medicaid vs other). Race and ethnicity were included as a social construct due to the differences in prevalence of cannabis use by race and ethnicity.

Alcohol and other substance use were assessed via the TWCQ and included alcohol (“During the past year, did you drink alcohol?” [yes or no]) and other substance use (“During the past year, did you use any other substance to get high, calm down, or stay awake?” [yes or no]). History of disruptive behavior disorders prior to an adolescent’s index date was determined using *ICD-9-CM* and *ICD-10-CM* codes (eMethods in Supplement 1). Disruptive behavior disorders were included as a covariate in sensitivity analyses to capture potential preexisting psychiatric conditions that might be coded as a disruptive behavior disorder but reflect underlying or prodromal mood or psychiatric disorders that are less consistently diagnosed in adolescents.

### Statistical Analysis

We fit extended Cox proportional hazard regression models using age as the time scale to estimate the association between time-varying cannabis use and incidence of each psychiatric disorder.^17^ Follow-up time began at the index cannabis screening, and adolescents were followed up until the outcome (incident psychiatric diagnosis) or censored at the earliest occurrence of membership disenrollment (>90-day gap), death, or the end of the study period (December 31, 2023). The maximum age at the end of follow-up was 25 years. All models were adjusted for sex, race and ethnicity, NDI, insurance type, and alcohol and other substance use. Cannabis, alcohol, and other substance use were modeled as time-varying covariates and were updated at each well-child visit. To account for shared genetic and environmental factors, we clustered adolescents within families using robust SEs.

We assessed the proportional hazards assumption for each model using weighted Schoenfeld residuals.^18^ For models with a departure from the proportional hazard assumption (ie, the association of cannabis use with the outcome was not constant over age), we estimated age interval–specific adjusted hazard ratios (AHRs) at 13 to 15 years, 16 to 17 years, 18 to 20 years, and 21 to 25 years. We also evaluated the potential implications of unmeasured confounding for our results by computing E-values for all model estimates and corresponding lower bounds of 95% CI limits.^19^

Given the complex and potentially bidirectional association between substance use and development of psychiatric disorders, we reran our main models after adjusting for history of other psychiatric disorders at baseline, including psychotic, bipolar, depressive, anxiety and disruptive behavior disorders, not including history of the outcome (eg, when incident psychotic disorders were the outcome, history of psychotic disorders was not included in the model). We repeated the analysis using the main models with a subset of the original sample after excluding adolescents with any history of psychiatric disorders at baseline. This conservative analysis examined whether associations would remain even among a lower-risk population of adolescents, where the likelihood of cannabis use starting before the onset of the outcomes of interest was higher. We also repeated the main analyses with 2 alternative definitions of psychotic disorders: (1) an expanded definition that additionally included *ICD-10* codes F10 to F19 substance use–induced psychotic disorders, and (2) a narrow version limited to unspecified psychosis not associated with substance use (*ICD-10* F29 codes; eMethods in Supplement 1).

All analyses were conducted from February 21, 2024, to August 27, 2025, using SAS v9.4 (SAS Institute, Inc.) Statistical significance was set at 2-sided *P* < .05.

## Results

The cohort included 463 396 adolescents (229 112 females [49.4%], 234 114 males [50.5%], 170 individuals [<0.1%] of other or unknown sex) with a mean (SD) age of 14.5 (1.3) years at baseline. Of these individuals, 136 708 (29.5%) were Hispanic, 93 737 (20.2%) were non-Hispanic Asian, 35 346 (7.6%) were non-Hispanic Black, 153 102 (33.0%) were non-Hispanic White, and 18 795 (4.1%) were multiracial or of other races or ethnicities (Table). At baseline, 26 345 adolescents (5.7%) self-reported cannabis use; 31 445 (6.8%), alcohol use; and 9872 (2.1%), other substance use. Adolescents reporting cannabis use were older, more likely to be female, Hispanic, non-Hispanic Black, and non-Hispanic White and less likely to be non-Hispanic Asian. Adolescents reporting cannabis use were more likely to have a higher NDI, Medicaid, and a higher prevalence of alcohol and other substance use.

**Table.** Sociodemographic Characteristics and Past-Year Cannabis Use at Baseline Among Participants

Characteristic	Adolescents, No. (%)	Baseline past-year cannabis use, No. (%)^a^	
		Yes	No
No. (%)	463 396 (100)	26 345 (5.7)	437 051 (94.3)
Age, mean (SD), y	14.5 (1.3)	15.6 (1.2)	14.4 (1.3)
Age, y^b^			
13	137 854 (29.7)	1616 (6.1)	136 238 (31.2)
14	134 955 (29.1)	4108 (15.6)	130 847 (29.9)
15	77 680 (16.8)	5291 (20.1)	72 389 (16.6)
16	62 197 (13.4)	7014 (26.6)	55 183 (12.6)
17	50 710 (10.9)	8316 (31.6)	42 394 (9.7)
Sex^b^			
Female	229 112 (49.4)	13 606 (51.6)	215 506 (49.3)
Male	234 114 (50.5)	12 721 (48.3)	221 393 (50.7)
Other or unknown^c^	170 (<0.0)	18 (0.1)	152 (<0.0)
Neighborhood deprivation index, quartile^b^			
First: least deprivation	97 025 (20.9)	4727 (17.9)	92 298 (21.1)
Second	131 906 (28.5)	7577 (28.8)	124 329 (28.4)
Third	126 303 (27.3)	7233 (27.5)	119 070 (27.2)
Fourth: most deprivation	107 989 (23.3)	6801 (25.8)	101 188 (23.2)
Missing	173 (<0.0)	7 (<0.0)	166 (<0.0)
Race and ethnicity^b^			
Hispanic	136 708 (29.5)	8460 (32.1)	128 248 (29.3)
Non-Hispanic Asian	93 737 (20.2)	1909 (7.2)	91 828 (21.0)
Non-Hispanic Black	35 346 (7.6)	2882 (10.9)	32 464 (7.4)
Non-Hispanic White	153 102 (33.0)	10 725 (40.7)	142 377 (32.6)
Non-Hispanic multiracial or other^d^	18 795 (4.1)	1078 (4.1)	17 717 (4.1)
Missing	25 708 (5.5)	1291 (4.9)	24 417 (5.6)
Insurance type			
Medicaid	79 191 (17.1)	5428 (20.6)	73 763 (16.9)
Other^e^	384 205 (82.9)	20 918 (79.4)	363 287 (83.1)
Noncannabis substance use^a^			
Alcohol use	31 445 (6.8)	16 949 (64.3)	14 496 (3.3)
Other substance use^f^	9872 (2.1)	5971 (22.7)	3901 (0.9)
History of disruptive behavior disorder	19 516 (4.2)	2290 (8.7)	17 226 (3.9)

^a^ Past-year cannabis, alcohol, and other substance use was determined by self-report.

^b^ Percentages may not total 100 due to rounding.

^c^ Other sex was not categorized further.

^d^ Other race and ethnicity included American Indian or Alaska Native and Native Hawaiian or Pacific Islander.

^e^ Other included commercial insurance and Medicare insurance.

^f^ Other substance use was not categorized further.

Mean (SD) follow-up time and age at the end of follow-up were similar across outcomes: psychotic disorder: 3.7 (2.5) years of follow-up to 18.6 (2.9) years of age; bipolar disorder: 3.7 (2.5) years of follow-up to 18.6 (2.9) years of age; depressive disorder: 3.3 (2.4) years of follow-up to 18.2 (2.9) years of age; anxiety disorder: 3.3 (2.4) years of follow-up to 18.2 (2.8) years of age. Most adolescents completed 1 or more questionnaires (1: 250 371 [54.0%], 2: 135 844 [29.3%]; ≥3: 77 181 [16.7%]). During follow-up, there were 4105 diagnoses of incident psychotic disorder (0.24 per 1000 person-years), 4061 diagnoses of bipolar disorder (0.24 per 1000 person-years), 62 137 diagnoses of depressive disorder (4.50 per 100 person-years), and 73 096 diagnoses of anxiety disorder (5.64 per 100 person-years; eTable 1 in Supplement 1). In adjusted models, cannabis use was associated with greater risk of incident psychotic disorders (AHR, 2.19; 95% CI, 1.97-2.42), bipolar disorder (AHR, 2.01; 95% CI, 1.82-2.22), depressive disorder (AHR, 1.34; 95% CI, 1.30-1.39), and anxiety disorder (AHR, 1.24; 95% CI, 1.21-1.28) (Figure).

**Figure.**
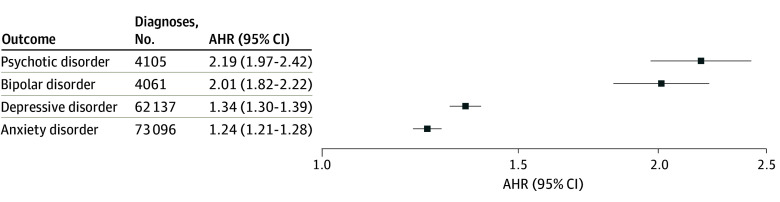
Forest Plot of Associations of Self-Reported Past-Year Cannabis Use Among 463 396 Adolescents Aged 13 to 17 Years and Incident Psychiatric Disorders Up to Age 26 Years Separate models were conducted for each of the 4 outcomes. All models were adjusted for sociodemographic characteristics and time-varying alcohol and other substance use. The x-axis is plotted on a logarithmic scale. AHR indicates adjusted hazard ratio.

There was a significant departure from the proportional hazards assumption for depressive and anxiety disorders, indicating that the AHRs declined with age (eFigure 1 in Supplement 1). For depressive disorder, the AHRs were 1.78 (95% CI, 1.68-1.89) at ages 13 to 15 years; 1.35 (95% CI, 1.30-1.41) at ages 16 to 17 years; 1.21 (95% CI, 1.15-1.27) at ages 18 to 20 years, and not statistically significant at ages 21 to 25 years (AHR, 0.97; 95% CI, 0.89-1.06). Similarly, for anxiety disorder, the AHRs were 1.47 (95% CI, 1.39-1.55) at ages 13 to 15 years; 1.24 (95% CI, 1.19-1.28) at ages 16 to 17 years, 1.24 (95% CI, 1.19-1.29) at ages 18 to 20 years, and not significant at ages 21 to 25 years (AHR, 0.95; 95% CI, 0.88-1.02).

### Sensitivity Analyses

Among those with an incident psychiatric disorder who reported cannabis use, the mean (SD) time elapsed from first report of past-year cannabis use to incident psychiatric disorder diagnosis ranged from 1.7 (1.6) to 2.3 (1.7) years (psychotic disorder: 2.0 [1.6] years; bipolar disorder: 2.3 [1.7] years; depressive disorder: 1.7 [1.6] years; anxiety disorder: 1.9 [1.6] years). After adjusting for other psychiatric disorders at baseline, associations of cannabis use and incident psychiatric disorders remained significant but were slightly attenuated (psychotic disorder AHR, 1.92; 95% CI, 1.73-2.13; bipolar disorder AHR, 1.73; 95% CI, 1.57-1.90; depressive disorder AHR, 1.33; 95% CI, 1.29-1.38; anxiety disorder AHR, 1.19; 95% CI, 1.16-1.23) (eFigure 2 in Supplement 1). In sensitivity analyses that excluded adolescents with any history of psychotic, bipolar, depressive, anxiety or disruptive behavior disorders at baseline, results were similar (psychotic disorder AHR, 1.99; 95% CI, 1.72-2.31; bipolar disorder AHR, 2.00; 95% CI, 1.73-2.30; depressive disorder AHR, 1.37; 95% CI, 1.32-1.42; anxiety disorder AHR, 1.22; 95% CI, 1.18-1.26 (eFigure 3 in Supplement 1). Sensitivity analyses examining associations of cannabis use and psychotic disorders using alternative outcome definitions yielded similar results (expanded definition AHR, 2.20; 95% CI, 1.99-2.44; narrow definition AHR, 2.21; 95% CI, 1.96-2.49) (eFigure 4 in Supplement 1).

### E-Values

For our main analyses, E-values ranged from 3.79 to 1.79 (psychotic disorder, 3.79; bipolar disorder, 3.44; depressive disorder, 2.02; anxiety disorder, 1.79) (eTable 2 in Supplement 1). This finding means that an unmeasured confounder would need to have associations of at least that range of magnitude with both past-year cannabis use and the outcome to fully explain the reported associations, conditional on measured covariates. In the sensitivity models, the results were generally similar but varied slightly across models (eTable 2 in Supplement 1).

## Discussion

In this cohort study of 463 396 adolescents followed up through age 25 years, past-year cannabis use was associated with an increased risk of incident psychotic, bipolar, depressive, and anxiety disorders. The strongest associations were found for psychotic and bipolar disorders, which is consistent with prior research suggesting that cannabis use during adolescence may be an especially strong risk factor for severe psychiatric outcomes.

These findings complement longitudinal epidemiologic studies that have found that adolescent cannabis use is associated with increased risk of psychotic experiences^20^ and adult psychotic disorders,^6^ as well as experimental studies that have reported an increase in psychotic experiences after intravenous tetrahydrocannabinol (THC) exposure.^21^ A recent population-based cohort study in Ontario, Canada, found that the risk of developing schizophrenia associated with CUD was greatest among adolescents and young adults.^7^ Furthermore, results are similar to findings from a meta-analysis of cannabis use and bipolar disorder, which found 2.63 times (95% CI, 1.95-3.53) greater odds of the emergence of bipolar disorder associated with cannabis use.^10^

Our results for depressive disorders are consistent with findings from prior meta-analyses that showed a higher risk of subsequent depression among those who used cannabis (odds ratio range, 1.17-1.37).^12,22,23^ However, our findings of increased risk for anxiety disorder differ from a meta-analysis that found no association between adolescent cannabis use and risk of anxiety during young adulthood.^2^ Notably, our study found that the strength of the associations between adolescent cannabis use and depressive and anxiety disorders decreased with age, and were no longer significant among young adults aged 21 to 25 years. This finding is consistent with other research suggesting that adolescence may be a particularly vulnerable period for cannabis-related psychopathology,^24^ and may partially explain inconsistent findings with anxiety outcomes.^2^ A recent population-based cohort study of children and adults with no previous health care visits for anxiety disorders in Ontario, Canada, found an increased incidence of anxiety-related emergency department visits or hospitalization following an incident emergency department visit for cannabis, with the most pronounced associations in younger individuals.^25^

Cannabis use during adolescence, particularly products containing high amounts of THC, could disrupt the endocannabinoid system at an important stage of development. THC acts on cannabinoid 1 receptors, which are highly expressed in the adolescent brain, and may disrupt neurodevelopment and affect areas of the brain associated with motivation, emotional, and affective processing, especially with early onset of cannabis use.^26^ In the present study, cannabis use among adolescents in northern California, where the typical average THC content of cannabis flower exceeds 20%,^27^ was associated with a 2.19 times greater risk of developing a psychotic disorder and 2.0 times greater risk of developing bipolar disorder by age 26 years. Given that daily cannabis use and use of higher-strength cannabis products may have a stronger association with psychiatric disorders,^2,4,6,9^ it is notable that we found associations even when defining cannabis solely as any past-year use.

Existing research suggests that the relationship between adolescent cannabis use and psychiatric disorders is complex and bidirectional. Adolescents with mental health symptoms or diagnoses may use cannabis as a way to mitigate distress, cannabis may contribute to mental health symptoms and diagnoses through neurobiological changes, and there may be shared social and biological risk factors that contribute to both mental health symptoms and cannabis use.^28^ Despite potential use of cannabis to self-medicate mental health symptoms, ongoing use of cannabis is associated with worsening mood symptoms^29^ and poorer adherence to medication treatment and psychotherapy.^30^ While it is not possible to definitively determine causality, this study had a strong retrospective cohort design. The temporal order of cannabis use preceded incident psychiatric disorder diagnoses by a mean of 1.7 to 2.3 years, supporting the possibility of a contributory role. The findings remained significant even after adjusting for a history of psychiatric disorders and other time-varying substance use and excluding adolescents with any history of a psychiatric disorder in sensitivity analyses, indicating unique associations between adolescent cannabis use and psychiatric disorders that go beyond broader adolescent psychopathology or substance use. This was a conservative approach, as other psychiatric disorders might be mediators or confounders depending on the timing and the underlying pathways. Furthermore, E-values indicated that only a strong unmeasured confounder with HRs of 3.79 to 1.79 could explain the associations, suggesting that adolescent cannabis use may be an independent risk factor. However, reverse causation cannot be ruled out, as some individuals may begin to use cannabis to self-medicate prodromal symptoms of psychiatric disorders even before a diagnosis is made. Future research with more nuanced measurement of cannabis use, including frequency, mode of use, and product strength, alongside regular screening and assessment for psychiatric disorder symptoms and diagnosis would help to further elucidate the timing and mechanisms underlying these associations.

Cannabis use was most common among older adolescents; Hispanic, non-Hispanic Black, and non-Hispanic White adolescents; and those with Medicaid or living in neighborhoods with a higher NDI. The overall results and these risk factors for health disparities reinforce the importance of early screening for cannabis use during pediatric visits and suggest that public health programs aimed at preventing or delaying adolescent cannabis use could hold promise for mitigating risk for the development of future psychiatric disorders. Increasing evidence on the associations of adolescent cannabis use with increased risk of developing psychiatric disorders could inform more effective policies to limit youth access, marketing, and exposure in legal cannabis markets through more prominent health warnings; stricter enforcement of sales to minors; stricter packaging, flavor, and advertising restrictions; and more targeted prevention, intervention, and mental health referral efforts to mitigate disparities.

### Strengths and Limitations

Our study has several strengths, including a large, sociodemographically diverse sample of adolescents who were universally screened for cannabis use during a developmental period when use typically begins, reducing selection bias and avoiding retrospective reporting. Unlike most studies using population-based data that rely on CUD diagnoses, we measured any self-reported cannabis use on a large scale. The study had adequate power to assess associations with the incidence of severe but less common outcomes of bipolar and psychotic disorders, as well as more common depressive and anxiety disorders, including adjustment disorder with depressed mood. We adjusted for sociodemographics, psychiatric confounders, and time-varying other substance use. Our large sample size allowed us to examine age-specific differences, allowing us to more comprehensively address the implications of cannabis use for specific subgroups.

Our study has several limitations. The sample comprised adolescents in a large northern California health care system who were screened for cannabis use during standard pediatric care, which may limit generalizability to uninsured youth, those without regular care, or those in states where cannabis is not legal. Self-reported past-year cannabis use may underestimate actual use, and rates reported in this study are lower than rates among adolescents in the US. It is possible that those who use cannabis more frequently are more willing to report their use than those who rarely use it, and our findings could reflect consequences associated with more frequent use. Future research with more detailed measures (eg, mode, frequency, product strength) and urine toxicology testing is needed.

Psychiatric outcomes were based on diagnoses documented in the EHR; therefore, cases might have been missed if symptoms were unreported. However, universal mental health screening is standard pediatric care at KPNC, increasing the likelihood of diagnosis when one exists. Although cannabis use preceded clinician-documented outcomes in our analyses, unmeasured confounding (eg, adverse childhood experiences, genetic risk, parent mental health) cannot be ruled out. In addition, approximately two-thirds of psychotic disorder diagnoses were classified as unspecified psychosis not due to a substance or known physiologic condition, potentially reflecting cautious diagnostic practices in adolescent care and limiting our ability to assess whether associations differed across specific psychotic diagnoses. Finally, while we examined differences by age, future research is needed to examine how associations vary with other sociodemographic factors (eg, sex, race and ethnicity, and socioeconomic status).

## Conclusions

To our knowledge, this is among the largest longitudinal studies to examine cannabis use in adolescence and its association with the incidence of clinician-diagnosed psychiatric disorders into young adulthood. Using population-level, EHR-based clinical data, we found that adolescent cannabis use was associated with an increased risk of multiple psychiatric disorders by early adulthood. Associations were stronger for psychotic and bipolar disorders. Together, these results are consistent with cannabis being a risk factor for or exacerbating the risk of psychiatric disorders rather than only resulting from preexisting psychiatric conditions. These findings reinforce the need for early prevention efforts, stronger public health messaging, and policy strategies that limit youth exposure in the context of expanding cannabis legalization.

## Notes

Supplement 1. **eFigure 1.** Association of cannabis use and depressive and anxiety disorders among adolescents, by age group **eFigure 2.** Association of cannabis use and psychiatric disorders among adolescents, additionally adjusted for prior psychiatric disorders **eFigure 3.** Association of cannabis use and psychiatric disorders among adolescents, excluding adolescents with history of psychiatric disorders at baseline **eFigure 4.** Association of adolescent cannabis use and alternative definitions of psychotic disorders **eTable 1.** Baseline sociodemographic characteristics stratified by psychiatric disorder diagnosis in follow-up **eTable 2.** E-values for fully-adjusted extended Cox proportional hazards models estimating associations between adolescent cannabis use and incident psychiatric disorders
eMethods.

Supplement 2.
Data Sharing Statement
