# Supplementary material for: Adolescent Cannabis Use and Risk of Psychotic, Bipolar, Depressive, and Anxiety Disorders
Source: JAMA Health Forum. 2026 Feb 20;7(2):e256839. doi: 10.1001/jamahealthforum.2025.6839 (PMC12924094; doi:10.1001/jamahealthforum.2025.6839)
Supplement: Supplement 1. — eFigure 1. Association of cannabis use and depressive and anxiety disorders among adolescents, by age group eFigure 2. Association of cannabis use and psychiatric disorders among adolescents, additionally adjusted for prior psychiatric disorders eFigure 3. Association of cannabis use and psychiatric disorders among adolescents, excluding adolescents with history of psychiatric disorders at baseline eFigure 4. Association of adolescent cannabis use and alternative definitions of psychotic disorders eTable 1. Baseline sociodemographic characteristics stratified by psychiatric disorder diagnosis in follow-up eTable 2. E-values for fully-adjusted extended Cox proportional hazards models estimating associations between adolescent cannabis use and incident psychiatric disorders eMethods. [file jamahealthforum-e256839-s001.pdf]

Supplemental Online Content

Young-Wolff KC, Cortez CA, Alexeeff SE, et al. Adolescent cannabis use and risk of psychotic, bipolar, depressive, and anxiety disorders. *JAMA Health Forum.* 2026;7(2): e256839. doi: 10.1001/jamahealthforum.2025.6839

eFigure 1. Association of cannabis use and depressive and anxiety disorders among adolescents, by age group

eFigure 2. Association of cannabis use and psychiatric disorders among adolescents, additionally adjusted for prior psychiatric disorders

eFigure 3. Association of cannabis use and psychiatric disorders among adolescents, excluding adolescents with history of psychiatric disorders at baseline

eFigure 4. Association of adolescent cannabis use and alternative definitions of psychotic disorders

eTable 1. Baseline sociodemographic characteristics stratified by psychiatric disorder diagnosis in follow-up

eTable 2. E-values for fully-adjusted extended Cox proportional hazards models estimating associations between adolescent cannabis use and incident psychiatric disorders

eMethods.

This supplemental material has been provided by the authors to give readers additional information about their work.

© 2026 Young-Wolff KC et al. *JAMA Health Forum*.

eFigure 1. Association of cannabis use and depressive and anxiety disorders among adolescents, by age group

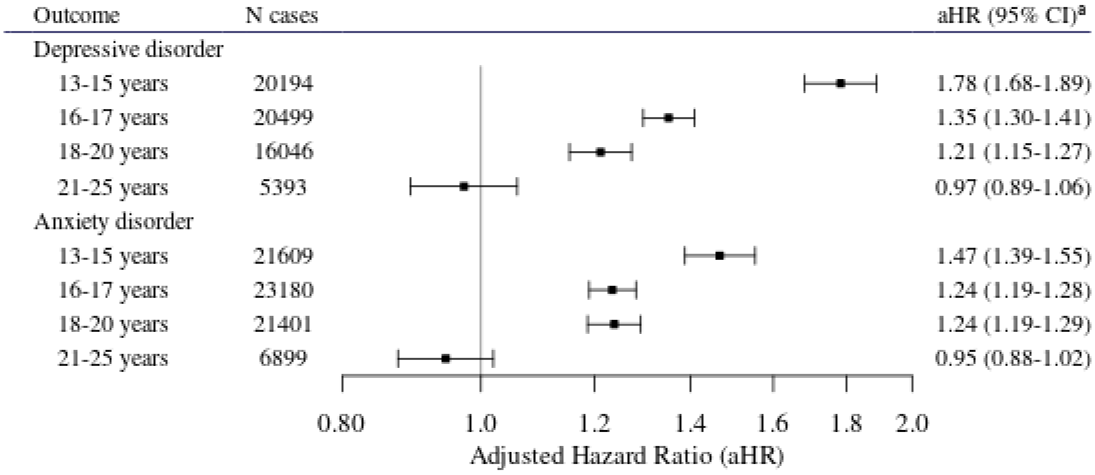

X-axis is plotted on the logarithmic scale.

[a]Two separate models were conducted specifying depressive and anxiety disorders as outcomes, partitioned by age group. Models were adjusted for sociodemographic characteristics and alcohol and other substance use.

© 2026 Young-Wolff KC et al. *JAMA Health Forum*.

eFigure 2. Association of cannabis use and psychiatric disorders among adolescents, additionally adjusted for prior psychiatric disorders

a. Association of cannabis use and psychiatric disorders among adolescents, additionally adjusted for prior psychiatric disorders

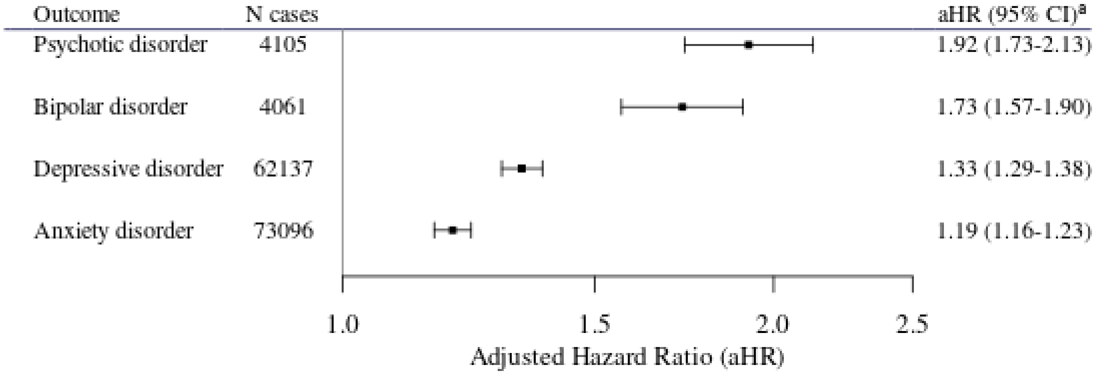

X-axis is plotted on the logarithmic scale.

[a]Four separate models were conducted specifying psychotic, depressive, bipolar, and anxiety disorders as outcomes. All models were adjusted for sociodemographic characteristics, alcohol and other substance use, and prior psychiatric disorders (disruptive behavior and other psychiatric disorders).

b. Association of cannabis use and depressive disorder among adolescents, additionally adjusted for prior psychiatric disorders, by age group

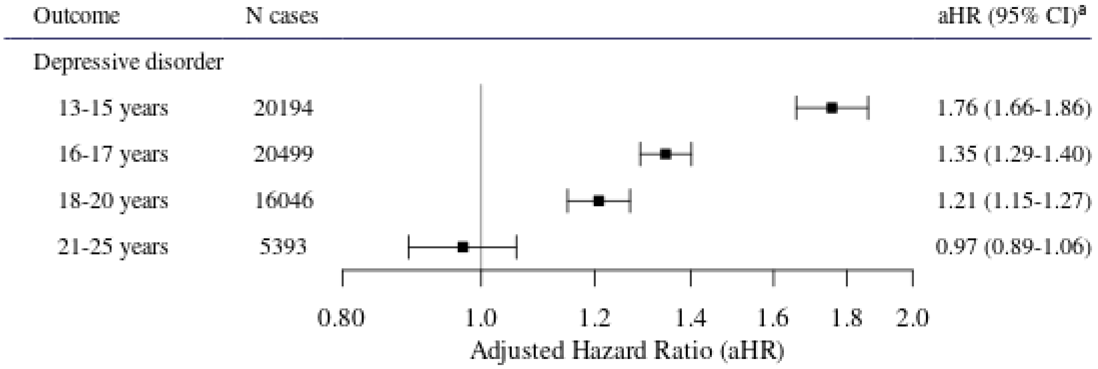

X-axis is plotted on the logarithmic scale.

[a]Model specifies depressive disorder as the outcome and is partitioned by age group, adjusted for sociodemographic characteristics, alcohol and other substance use, and prior psychiatric disorders (disruptive behavior and other psychiatric disorders).

© 2026 Young-Wolff KC et al. *JAMA Health Forum*.

eFigure 3. Association of cannabis use and psychiatric disorders among adolescents, excluding adolescents with history of psychiatric disorders at baseline

a. Association of cannabis use and psychiatric disorders among adolescents, excluding adolescents with history of psychiatric disorders at baseline

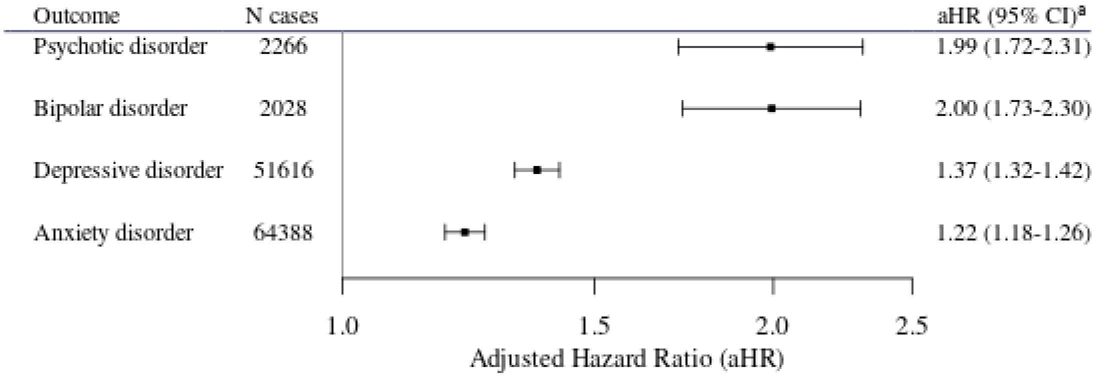

X-axis is plotted on the logarithmic scale.

[a]Four separate models were conducted specifying psychotic, depressive, bipolar, and anxiety disorders as outcomes. All models were adjusted for sociodemographic characteristics, and alcohol and other substance use.

© 2026 Young-Wolff KC et al. *JAMA Health Forum*.

b. Association of cannabis use and incident psychiatric disorders, excluding adolescents with history of psychiatric disorders at baseline, by age group

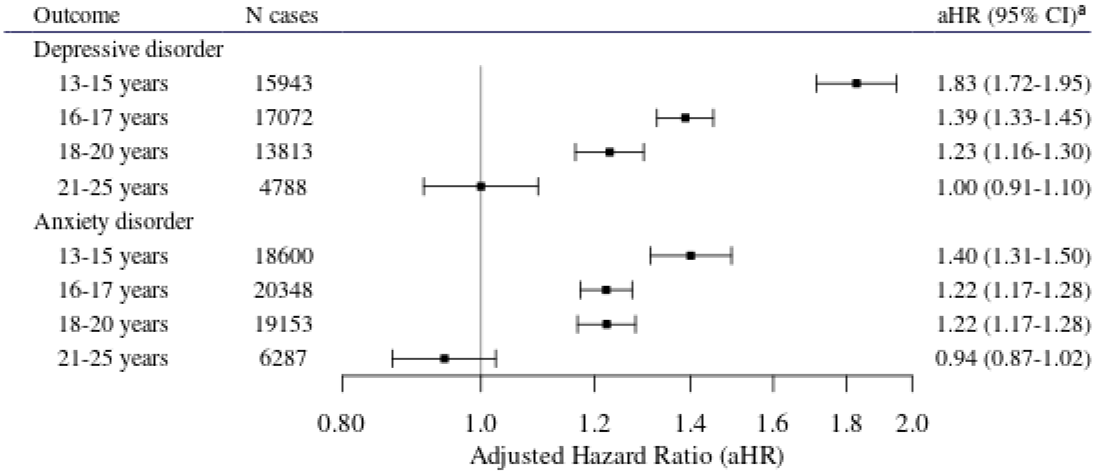

X-axis is plotted on the logarithmic scale.

[a]Two separate models were conducted specifying depressive and anxiety disorders as outcomes, partitioned by age group. Models were adjusted for sociodemographic characteristics and alcohol and other substance use.

eFigure 4. Association of adolescent cannabis use and alternative definitions of psychotic disorders

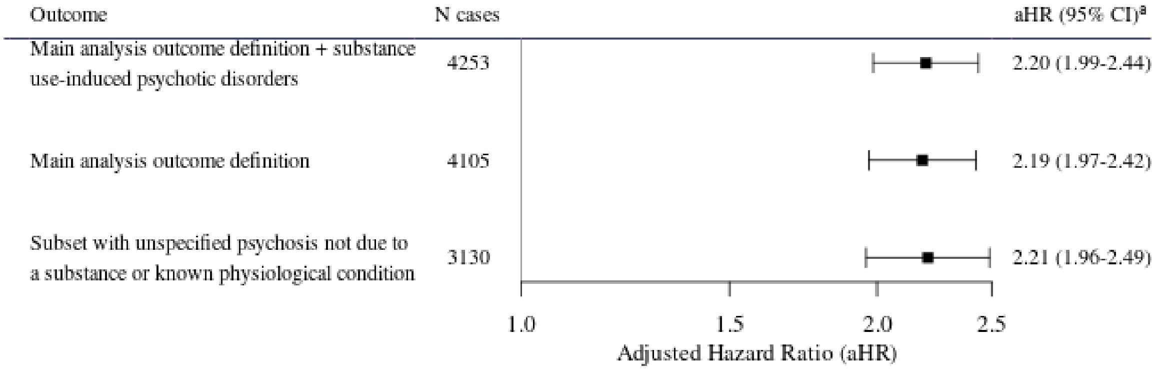

X-axis is plotted on the logarithmic scale.

[a]In addition to the main psychotic disorder analysis, two separate models were conducted specifying an expanded definition of psychotic disorder (main analysis outcome definition + substance use-induced psychotic disorders) and a narrow definition (unspecified psychosis not due to a substance or known physiological condition). Models were adjusted for sociodemographic characteristics and alcohol and other substance use.

© 2026 Young-Wolff KC et al. *JAMA Health Forum*.

eTable 1. Baseline sociodemographic characteristics stratified by psychiatric disorder diagnosis in follow-up

	Any psychiatric disorder diagnosis in follow-up		Psychiatric disorder diagnosis in follow-up			
**Characteristic, no. (%)**	**Yes N = 98,396**	**No N = 365,000**	**Psychotic disorder N = 4,105**	**Bipolar disorder N = 4,061**	**Depressive disorder N = 62,137**	**Anxiety disorder N = 73,096**
Age, years, mean (SD)	14.6 (1.4)	14.4 (1.3)	14.8 (1.4)	15 (1.4)	14.5 (1.3)	14.6 (1.4)
Age, years						
13	26,722 (27.2)	111,132 (30.4)	924 (22.5)	775 (19.1)	17,571 (28.3)	19,557 (26.8)
14	27,059 (27.5)	107,896 (29.6)	1,087 (26.5)	962 (23.7)	17,497 (28.2)	19,837 (27.1)
15	17,570 (17.9)	60,110 (16.5)	721 (17.6)	728 (17.9)	11,108 (17.9)	13,138 (18.0)
16	14,903 (15.1)	47,294 (13.0)	743 (18.1)	824 (20.3)	8,933 (14.4)	11,259 (15.4)
17	12,142 (12.3)	38,568 (10.6)	630 (15.3)	772 (19.0)	7,028 (11.3)	9,305 (12.7)
Sex						
Female	63,098 (64.1)	166,014 (45.5)	1,942 (47.3)	2,720 (67.0)	40,110 (64.6)	48,210 (66.0)
Male	35,222 (35.8)	198,892 (54.5)	2,156 (52.5)	1,333 (32.8)	21,978 (35.4)	24,831 (34.0)
Other or unknown	76 (0.1)	94 (0.0)	7 (0.2)	8 (0.2)	49 (0.1)	55 (0.1)
Neighborhood deprivation, quartile						
Q1: least deprivation	19,925 (20.2)	77,100 (21.1)	719 (17.5)	863 (21.3)	12,280 (19.8)	14,976 (20.5)
Q2	29,637 (30.1)	102,269 (28.0)	1,197 (29.2)	1,269 (31.2)	18,739 (30.2)	22,096 (30.2)
Q3	26,883 (27.3)	99,420 (27.2)	1,149 (28.0)	1,092 (26.9)	17,066 (27.5)	19,954 (27.3)
Q4: most deprivation	21,915 (22.3)	86,074 (23.6)	1,040 (25.3)	837 (20.6)	14,027 (22.6)	16,043 (21.9)
Missing	36 (0.0)	137 (0.0)	0 (0.0)	0 (0.0)	25 (0.0)	27 (0.0)
Race and ethnicity						
Hispanic	29,039 (29.5)	107,669 (29.5)	1,184 (28.8)	976 (24.0)	18,119 (29.2)	21,584 (29.5)
Non-Hispanic						
Asian	15,191 (15.4)	78,546 (21.5)	558 (13.6)	512 (12.6)	10,040 (16.2)	11,615 (15.9)
Black	7,823 (8.0)	27,523 (7.5)	536 (13.1)	413 (10.2)	5,222 (8.4)	5,455 (7.5)
Multiple or other[b]	3,948 (4.0)	14,847 (4.1)	210 (5.1)	168 (4.1)	2,555 (4.1)	2,921 (4.0)
White	38,388 (39.0)	114,714 (31.4)	1,498 (36.5)	1,878 (46.2)	23,768 (38.3)	28,476 (39.0)
Missing	4,007 (4.1)	21,701 (5.9)	119 (2.9)	114 (2.8)	2,433 (3.9)	3,045 (4.2)
Insurance type						
Medicaid	16,513 (16.8)	62,678 (17.2)	759 (18.5)	732 (18.0)	10,293 (16.6)	11,874 (16.2)

© 2026 Young-Wolff KC et al. *JAMA Health Forum*.

Characteristic, no. (%)	Any psychiatric disorder diagnosis in follow-up		Psychiatric disorder diagnosis in follow-up			
	Yes N = 98,396	No N = 365,000	Psychotic disorder N = 4,105	Bipolar disorder N = 4,061	Depressive disorder N = 62,137	Anxiety disorder N = 73,096
Other	81,883 (83.2)	302,322 (82.8)	3,346 (81.5)	3,329 (82.0)	51,844 (83.4)	61,222 (83.8)
Non-cannabis substance use[a]						
Alcohol use	9,349 (9.5)	22,096 (6.1)	615 (15.0)	793 (19.5)	5,494 (8.8)	6,663 (9.1)
Other substance use	3,098 (3.1)	6,774 (1.9)	307 (7.5)	347 (8.5)	1,801 (2.9)	2,025 (2.8)
History of disruptive behavior disorder	4,406 (4.5)	15,110 (4.1)	496 (12.1)	479 (11.8)	2,491 (4.0)	2,450 (3.4)

[a]Past-year cannabis, alcohol, and other substance use was determined by self-report.

[b]Multiple or other race and ethnicity includes American Indian, Alaska Native, Pacific Islander, Native Hawaiian, and multiracial individuals.

© 2026 Young-Wolff KC et al. *JAMA Health Forum*.

eTable 2. E-values for fully-adjusted extended Cox proportional hazards models estimating associations between adolescent cannabis use and incident psychiatric disorders

a. E-values for fully-adjusted extended Cox proportional hazards models estimating associations between adolescent cannabis use and incident psychiatric disorders

	Cannabis use	
Outcome	e-value for aHR	e-value for CI
Psychotic disorder	3.79	3.35
Bipolar disorder	3.44	3.05
Depressive disorder	2.02	1.92
Anxiety disorder	1.79	1.70

Notes: aHR = adjusted Hazards Ratio. CI = 95% confidence interval.

b. E-values for fully-adjusted extended Cox proportional hazards models estimating associations between adolescent cannabis use and incident depressive and anxiety disorder, by age group

	Cannabis use	
Outcome	e-value for aHR	e-value for CI
Depressive disorder		
13-15 years	2.96	2.76
16-17 years	2.04	1.92
18-20 years	1.72	1.57
21-25 years	1.20	1.00
Anxiety disorder		
13-15 years	2.30	2.12
16-17 years	1.77	1.67
18-20 years	1.78	1.66
21-25 years	1.31	1.00

Notes: aHR = adjusted Hazards Ratio. CI = 95% confidence interval.

c. E-values for fully-adjusted extended Cox proportional hazards models estimating associations between adolescent cannabis use and incident psychiatric disorders, additionally adjusted for prior psychiatric disorders, including disruptive behavior disorders.

	Cannabis use	
Outcome	e-value for aHR	e-value for CI
Psychotic disorder	3.25	2.86
Bipolar disorder	2.85	2.51
Depressive disorder	2.00	1.91
Anxiety disorder	1.68	1.59

Notes: aHR = adjusted Hazards Ratio. CI = 95% confidence interval.

© 2026 Young-Wolff KC et al. *JAMA Health Forum*.

d. E-values for fully-adjusted extended Cox proportional hazards models estimating associations between adolescent cannabis use and incident depressive disorder, additionally adjusted for prior psychiatric disorders, including disruptive behavior disorders, by age group

	Cannabis use	
Outcome	e-value for aHR	e-value for CI
Depressive disorder		
13-15 years	2.91	2.70
16-17 years	2.03	1.90
18-20 years	1.71	1.56
21-25 years	1.21	1.00

Notes: aHR = adjusted Hazards Ratio. CI = 95% confidence interval.

e. E-values for fully-adjusted extended Cox proportional hazards models estimating associations between adolescent cannabis use and incident psychiatric disorders among adolescents, excluding adolescents with history of psychiatric disorders at baseline

	Cannabis use	
Outcome	e-value for aHR	e-value for CI
Psychotic disorder	3.40	2.83
Bipolar disorder	3.40	2.85
Depressive disorder	2.08	1.97
Anxiety disorder	1.73	1.64

Notes: aHR = adjusted Hazards Ratio. CI = 95% confidence interval.

f. E-values for fully-adjusted extended Cox proportional hazards models estimating associations between adolescent cannabis use and incident depressive and anxiety disorders, excluding adolescents with history of psychiatric disorders at baseline, by age group

	Cannabis use	
Outcome	e-value for aHR	e-value for CI
Depressive disorder		
13-15 years	3.06	2.82
16-17 years	2.12	1.99
18-20 years	1.76	1.61
21-25 years	1.00	1.00
Anxiety disorder		
13-15 years	2.15	1.95
16-17 years	1.75	1.62
18-20 years	1.75	1.61
21-25 years	1.31	1.00

Notes: aHR = adjusted Hazards Ratio. CI = 95% confidence interval.

© 2026 Young-Wolff KC et al. *JAMA Health Forum*.

g. E-values for fully-adjusted extended Cox proportional hazards models estimating associations between adolescent cannabis use and alternative definitions of incident psychotic disorders

	Cannabis use	
Outcome definition	e-value for aHR	e-value for CI
Main analysis outcome definition + substance use-induced psychotic disorders	3.82	3.39
Main analysis outcome definition	3.79	3.35
Subset with unspecified psychosis not due to a substance or known physiological condition	3.84	3.33

Notes: aHR = adjusted Hazards Ratio. CI = 95% confidence interval.

© 2026 Young-Wolff KC et al. *JAMA Health Forum*.

eMethods.

a. STROBE Diagram of Patient Selection

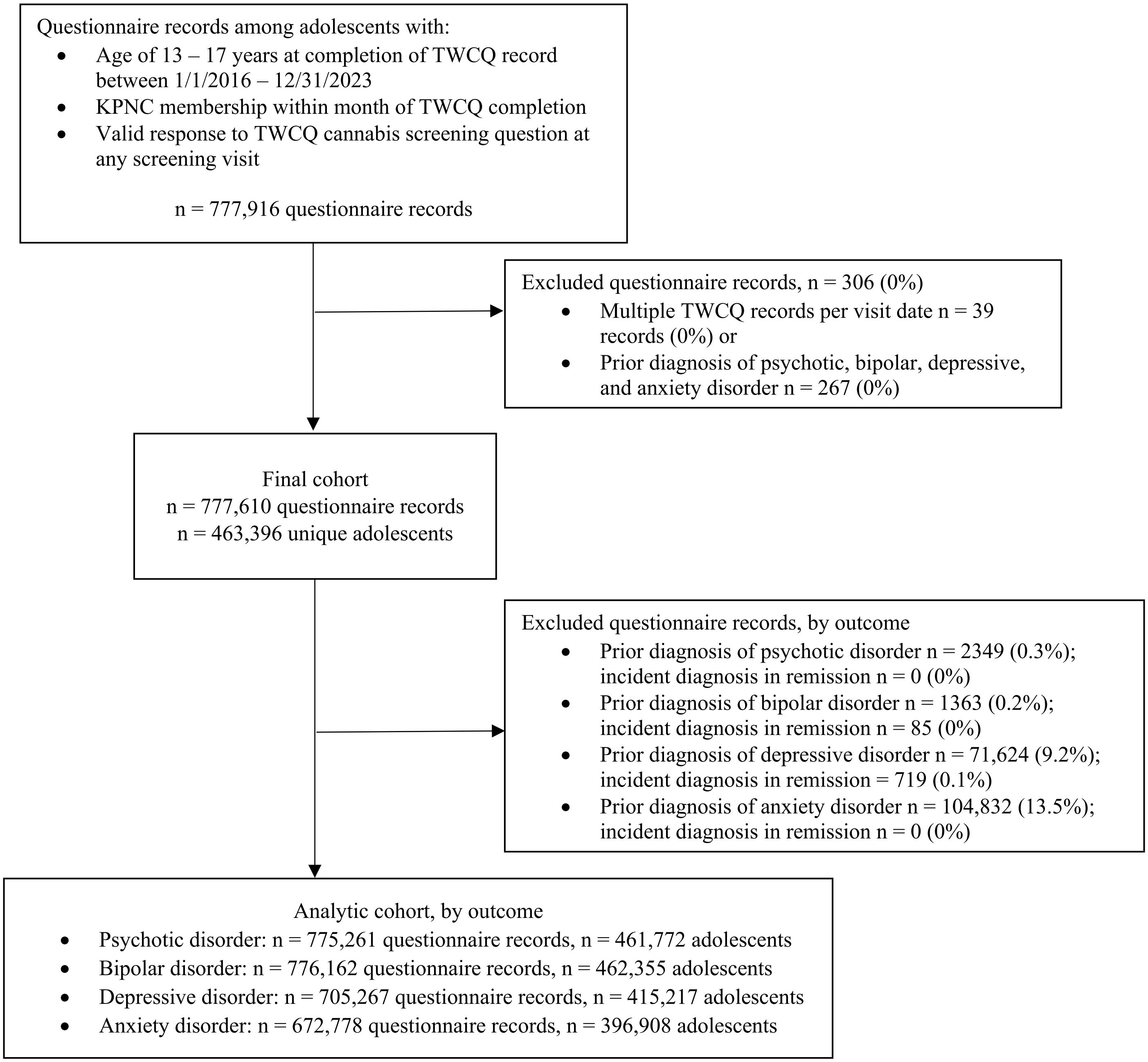

Abbreviation: KPNC, Kaiser Permanente Northern California; TWCQ, Teen Well Check Questionnaire

© 2026 Young-Wolff KC et al. *JAMA Health Forum*.

b. ICD-9-CM and ICD-10-CM diagnosis codes used to identify anxiety, bipolar, depressive, psychotic, and disruptive behavior disorders

Anxiety disorder

ICD-9-CM: 300.0*, 300.21, 300.3, 309.21, 309.24, 309.28

ICD-10-CM: F41*, F42*, F43.22, F43.23

Bipolar disorder

ICD-9-CM: 296.0*, 296.1*, 296.4*, 296.5*, 296.6*, 296.80, 296.81, 296.89, 301.13

ICD-10-CM: F30*, F31*, F34.0

Depressive disorder

ICD-9-CM: 293.83, 296.2*, 296.3*, 296.82, 293.83, 296.9*, 300.4, 301.12, 309.0, 309.1, 309.28, 311, 799.24

ICD-10-CM: F32*, F33*, F34.1, F34.8, F34.89, F34.9, F43.21, F43.23, R45.86

Psychotic disorder

ICD-9-CM: 292.1*, 295*, 297*, 298*

ICD-10-CM: F20*, F22, F23, F25*, F28, F29, F10.15*, F10.25*, F10.95*, F11.15*, F11.25*, F11.95*, F12.15*, F12.25*, F12.95*, F13.15*, F13.25*, F13.95*, F14.15*, F14.25*, F14.95*, F15.15*, F15.25*, F15.95*, F16.15*, F16.25*, F16.95*, F18.15*, F18.25*, F18.95*, F19.15*, F19.25*, F19.95*

Disruptive behavior disorders

ICD-9-CM: 301.7, 312*, 313.81

ICD-10-CM: F60.2, F63*, F91*

© 2026 Young-Wolff KC et al. *JAMA Health Forum*.

c. Summary of ICD-10-CM diagnosis codes among adolescents with incident psychiatric disorders

Psychotic disorder

Code	Description	N (%)
F29	Unspecified psychosis not due to a substance or known physiological condition	3130 (76.2)
F23	Brief psychotic disorder	319 (7.8)
F22	Delusional disorders	260 (6.3)
F20.9	Schizophrenia, unspecified	147 (3.6)
F28	Other psychotic disorder not due to a substance or known physiological condition	61 (1.5)
F25.9	Schizoaffective disorder, unspecified	54 (1.3)
F25.0	Schizoaffective disorder, bipolar type	51 (1.2)
F25.1	Schizoaffective disorder, depressive type	34 (0.8)
F20.81	Schizophreniform disorder	23 (0.6)
F20.2	Catatonic schizophrenia	11 (0.3)
F21	Schizotypal disorder	11 (0.3)
F20.89	Other schizophrenia	3 (0.1)
F20.3	Undifferentiated schizophrenia	1 (0)

Bipolar disorder

Code	Description	N (%)
F31.9	Bipolar disorder, unspecified	1584 (39)
F31.81	Bipolar II disorder	1131 (27.9)
F30.9	Manic episode, unspecified	259 (6.4)
F31.2	Bipolar disorder, current episode manic severe with psychotic features	179 (4.4)
F31.89	Other bipolar disorder	153 (3.8)
F34.0	Cyclothymic disorder	138 (3.4)
F31.32	Bipolar disorder, current episode depressed, moderate	80 (2)
F31.4	Bipolar disorder, current episode depressed, severe, without psychotic features	77 (1.9)
F31.5	Bipolar disorder, current episode depressed, severe, with psychotic features	74 (1.8)
F30.2	Manic episode, severe with psychotic symptoms	73 (1.8)
F31.10	Bipolar disorder, current episode manic without psychotic features, unspecified	38 (0.9)
F31.0	Bipolar disorder, current episode hypomanic	35 (0.9)
F31.31	Bipolar disorder, current episode depressed, mild	32 (0.8)

© 2026 Young-Wolff KC et al. *JAMA Health Forum*.

Code	Description	N (%)
F31.30	Bipolar disorder, current episode depressed, mild or moderate severity, unspecified	30 (0.7)
F31.60	Bipolar disorder, current episode mixed, unspecified	29 (0.7)
F31.64	Bipolar disorder, current episode mixed, severe, with psychotic features	29 (0.7)
F31.12	Bipolar disorder, current episode manic without psychotic features, mod	28 (0.7)
F31.11	Bipolar disorder, current episode manic without psychotic features, mild	21 (0.5)
F31.63	Bipolar disorder, current episode mixed, severe, without psychotic features	16 (0.4)
F30.8	Other manic episodes	15 (0.4)
F31.13	Bipolar disorder, current episode manic without psychotic features, severe	15 (0.4)
F31.62	Bipolar disorder, current episode mixed, moderate	13 (0.3)
F30.10	Manic episode without psychotic symptoms, unspecified	4 (0.1)
F30.11	Manic episode without psychotic symptoms, mild	3 (0.1)
F31.61	Bipolar disorder, current episode mixed, mild	3 (0.1)
F30.12	Manic episode without psychotic symptoms, moderate	1 (0)
F30.13	Manic episode, severe, without psychotic symptoms	1 (0)

Depressive disorder

Code	Description	N (%)
F32.9	Major depressive disorder, single episode, unspecified	17374 (28)
F43.23	Adjustment disorder with mixed anxiety and depressed mood	12235 (19.7)
F32.A	Depression, unspecified	9011 (14.5)
F43.21	Adjustment disorder with depressed mood	5641 (9.1)
F33.1	Major depressive disorder, recurrent, moderate	5104 (8.2)
F32.1	Major depressive disorder, single episode, moderate	4644 (7.5)
F34.1	Dysthymic disorder	1675 (2.7)
F32.0	Major depressive disorder, single episode, mild	1478 (2.4)
F33.0	Major depressive disorder, recurrent, mild	1449 (2.3)
F33.2	Major depressive disorder, recurrent severe without psychotic features	1441 (2.3)
F32.2	Major depressive disorder, single episode, severe without psychotic features	1152 (1.9)
F34.89	Other specified persistent mood disorders	364 (0.6)
F33.9	Major depressive disorder, recurrent, unspecified	231 (0.4)
F33.3	Major depressive disorder, recurrent, severe with psychotic symptoms	117 (0.2)
F32.3	Major depressive disorder, single episode, severe with psychotic features	81 (0.1)

© 2026 Young-Wolff KC et al. *JAMA Health Forum*.

Code	Description	N (%)
R45.86	Emotional lability	55 (0.1)
F32.8	Other depressive episodes	31 (0)
F33.8	Other recurrent depressive disorders	25 (0)
F34.8	Other persistent mood [affective] disorders	15 (0)
F34.9	Persistent mood [affective] disorder, unspecified	14 (0)

Anxiety disorder

Code	Description	N (%)
F41.9	Anxiety disorder, unspecified	39662 (54.3)
F43.23	Adjustment disorder with mixed anxiety and depressed mood	10698 (14.6)
F41.1	Generalized anxiety disorder	9075 (12.4)
F41.8	Other specified anxiety disorders	5799 (7.9)
F43.22	Adjustment disorder with anxiety	3844 (5.3)
F41.0	Panic disorder without agoraphobia	3136 (4.3)
F42.9	Obsessive-compulsive disorder, unspecified	765 (1)
F42.8	Other obsessive-compulsive disorder	29 (0)
F93.0	Separation anxiety disorder of childhood	29 (0)
F42.2	Mixed obsessional thoughts and acts	28 (0)
F42	Obsessive-compulsive disorder	26 (0)
F41.3	Other mixed anxiety disorders	3 (0)
F40.01	Agoraphobia, with panic disorder	2 (0)

© 2026 Young-Wolff KC et al. *JAMA Health Forum*.

d. Combinations of psychiatric disorder diagnoses in follow-up among adolescents with incident psychiatric anxiety, bipolar, depressive, and psychotic disorders

Number of incident psychiatric disorders	Psychotic Disorder N = 4105 (100)		Bipolar Disorder N = 4061 (100)		Depressive Disorder N = 62137 (100)		Anxiety Disorder N=73096 (100)	
		N (%)		N (%)		N (%)		N (%)
1	Psychotic Disorder only	1007 (24.5)	Bipolar Disorder only	1041 (25.6)	Depressive Disorder only	21884 (35.2)	Incident Anxiety Disorder only	32738 (44.8)
2	Psychotic & Depressive Disorder	492 (12)	Bipolar & Depressive Disorder	398 (9.8)	Depressive & Bipolar Disorder	398 (0.6)	Anxiety & Depressive Disorder	36690 (50.2)
	Psychotic & Anxiety Disorder	484 (11.8)	Bipolar & Anxiety Disorder	478 (11.8)	Depressive & Anxiety Disorder	36690 (59)	Anxiety & Bipolar Disorder	478 (0.7)
	Psychotic & Bipolar Disorder	318 (7.7)	Bipolar & Psychotic Disorder	318 (7.8)	Depressive & Psychotic Disorder	492 (0.8)	Anxiety & Psychotic Disorder	484 (0.7)
3	Psychotic, Depressive, & Anxiety Disorder	1040 (25.3)	Bipolar, Depressive, & Anxiety Disorder	1062 (26.2)	Depressive, Bipolar, & Anxiety Disorder	1062 (1.7)	Anxiety, Depressive & Bipolar Disorder	1062 (1.5)
	Psychotic, Depressive, & Bipolar Disorder	160 (3.9)	Bipolar, Depressive, & Psychotic Disorder	160 (3.9)	Depressive, Anxiety & Psychotic Disorder	1040 (1.7)	Anxiety, Depressive & Psychotic Disorder	1040 (1.4)
	Psychotic, Bipolar, Anxiety, & Disorder	193 (4.7)	Bipolar, Anxiety, & Psychotic Disorder	193 (4.8)	Depressive, Bipolar, & Psychotic Disorder	160 (0.3)	Anxiety, Bipolar & Psychotic Disorder	193 (0.3)
4	All four psychiatric disorders	411 (10)	All four psychiatric disorders	411 (10.1)	All four psychiatric disorders	411 (0.7)	All four psychiatric disorders	411 (0.6)
	Total	4105 (100)	Total	4061 (100)	Total	62137 (100)	Total	73096 (100)

© 2026 Young-Wolff KC et al. *JAMA Health Forum*.

e. Summary of ICD-10-CM diagnosis codes among adolescents with incident psychotic disorders, by alternative outcome definitions

Outcome definition			Code	Description	N (%)
Expanded outcome definition Main analysis outcome definition + substance use-induced psychotic disorders			F12.15*, F12.25*, F12.95*	Cannabis abuse, dependence, or unspecified use with psychotic disorder	259 (6.4)
			F19.15*, F19.25*, F19.95*	Other abuse, dependence, or unspecified use substance dependence with psychoactive substance-induced psychotic disorder	172 (3.2)
			F10.15*, F10.25*, F10.95*	Alcohol abuse, dependence, or unspecified use with alcohol-induced psychotic disorder	19 (0)
			F15.15*, F15.25*, F15.95*	Other stimulant abuse, dependence, or unspecified use with stimulant-induced psychotic disorder	66 (0)
			F16.15*, F16.25*, F16.95*	Hallucinogen abuse, dependence, or unspecified use with hallucinogen-induced psychotic disorder	29 (0)
			F14.15*, F14.25*, F14.95*	Cocaine abuse, dependence, or unspecified use with cocaine-induced psychotic disorder	7 (0)
			F13.15*, F13.25*, F13.95*	Sedative, hypnotic or anxiolytic abuse, dependence, or unspecified use with sedative, hypnotic or anxiolytic-induced psychotic disorder	4 (0)
			F11.15*, F11.25*, F11.95*	Opioid abuse, dependence, or unspecified use with opioid-induced psychotic disorder	2 (0)
			F18.15*, F18.25*, F18.95*	Inhalant abuse, dependence, or unspecified use with inhalant-induced psychotic disorder	1 (0)
	Main analysis outcome definition Psychotic disorder		F23	Brief psychotic disorder	284 (6.4)
			F22	Delusional disorders	224 (6.4)
			F20.9	Schizophrenia, unspecified	126 (3.2)
			F28	Other psychotic disorder not due to a substance or known physiological condition	56 (0)
			F25.9	Schizoaffective disorder, unspecified	50 (0)
			F25.0	Schizoaffective disorder, bipolar type	50 (0)
			F25.1	Schizoaffective disorder, depressive type	33 (0)
			F20.81	Schizophreniform disorder	19 (0)
			F20.2	Catatonic schizophrenia	10 (0)
			F21	Schizotypal disorder	11 (0)
			F20.89	Other schizophrenia	3 (0)
			F20.3	Undifferentiated schizophrenia	1 (0)
		Narrow outcome definition	F29	Unspecified psychosis not due to a substance or known physiological condition	2823 (67.2)

© 2026 Young-Wolff KC et al. *JAMA Health Forum*.
