# Supplementary material for: Adolescent Cannabis Use and Risk of Psychotic, Bipolar, Depressive, and Anxiety Disorders
Source: JAMA Health Forum. 2026 Feb 20;7(2):e256839. doi: 10.1001/jamahealthforum.2025.6839 (PMC12924094; doi:10.1001/jamahealthforum.2025.6839)
Supplement: Supplement 2. — Data Sharing Statement [file jamahealthforum-e256839-s002.pdf]

Data Sharing Statement

Young-Wolff. Adolescent Cannabis Use and Risk of Psychotic, Bipolar, Depressive, and Anxiety Disorders. *JAMA Health Forum*. Published February 20, 2026.
doi:10.1001/jamahealthforum.2025.6839

Data
Data available: No
